# Comorbid Conditions in Idiopathic Pulmonary Fibrosis: Recognition and Management

**DOI:** 10.3389/fmed.2017.00123

**Published:** 2017-08-02

**Authors:** Justin M. Oldham, Harold R. Collard

**Affiliations:** ^1^Department of Medicine, Division of Pulmonary, Critical Care and Sleep Medicine, University of California at Davis, Davis, CA, United States; ^2^Department of Medicine, Division of Pulmonary and Critical Care Medicine, University of California at San Francisco, San Francisco, CA, United States

**Keywords:** idiopathic pulmonary fibrosis, idiopathic interstitial pneumonia, interstitial lung disease, pulmonary fibrosis, co-morbidity

## Abstract

Idiopathic pulmonary fibrosis (IPF), a fibrosing interstitial pneumonia of unknown etiology, primarily affects older adults and leads to a progressive decline in lung function and quality of life. With a median survival of 3–5 years, IPF is the most common and deadly of the idiopathic interstitial pneumonias. Despite the poor survivorship, there exists substantial variation in disease progression, making accurate prognostication difficult. Lung transplantation remains the sole curative intervention in IPF, but two anti-fibrotic therapies were recently shown to slow pulmonary function decline and are now approved for the treatment of IPF in many countries around the world. While the approval of these therapies represents an important first step in combatting of this devastating disease, a comprehensive approach to diagnosing and treating patients with IPF remains critically important. Included in this comprehensive assessment is the recognition and appropriate management of comorbid conditions. Though IPF is characterized by single organ involvement, many comorbid conditions occur within other organ systems. Common cardiovascular processes include coronary artery disease and pulmonary hypertension (PH), while gastroesophageal reflux and hiatal hernia are the most commonly encountered gastrointestinal disorders. Hematologic abnormalities appear to place patients with IPF at increased risk of venous thromboembolism, while diabetes mellitus (DM) and hypothyroidism are prevalent metabolic disorders. Several pulmonary comorbidities have also been linked to IPF, and include emphysema, lung cancer, and obstructive sleep apnea. While the treatment of some comorbid conditions, such as CAD, DM, and hypothyroidism is recommended irrespective of IPF, the benefit of treating others, such as gastroesophageal reflux and PH, remains unclear. In this review, we highlight common comorbid conditions encountered in IPF, discuss disease-specific diagnostic modalities, and review the current state of treatment data for several key comorbidities.

## Introduction

Idiopathic Pulmonary Fibrosis (IPF), a fibrosing interstitial lung disease (ILD) of unknown etiology, primarily affects older adults and leads to a progressive decline in lung function and quality of life (1–4). With an estimated prevalence of 18–63 cases per 100,000 and median survival of 3–5 years (5, 6), IPF remains the most common and deadly of the idiopathic interstitial pneumonias (IIPs). Despite poor survivorship, there exists substantial variability in disease progression, whereby most patients experience a steady clinical decline, some remain stable over many years and others die from rapidly progressive disease (3, 7, 8). Lung transplantation remains the sole curative intervention for IPF, but two anti-fibrotic therapies were recently shown to slow pulmonary function decline in phase III clinical trials (9–11). *Post hoc* analyses of clinical trial datasets also suggest that anti-fibrotic therapy may reduce the risk of acute exacerbations and improve overall survival in those with IPF (12–14).

While the identification of therapies that effectively slow IPF progression represents a monumental step forward in the care of patients with IPF, pharmacotherapy is but one component of the multi-pronged approach necessary to optimally manage patients with IPF. Other equally important pieces include evidence-based prognostication (15) improvement of functional status with formal pulmonary rehabilitation and supplemental oxygen (where appropriate), patient education with regard to IPF pathobiology, natural history, and clinical trial availability, and management of common comorbidities (4, 16). In this review, we highlight common comorbidities encountered in IPF, discuss diagnostic and screening modalities, and review the current state of treatment data for such conditions.

## Pulmonary Comorbidities

### Emphysema

**Figure 1:**
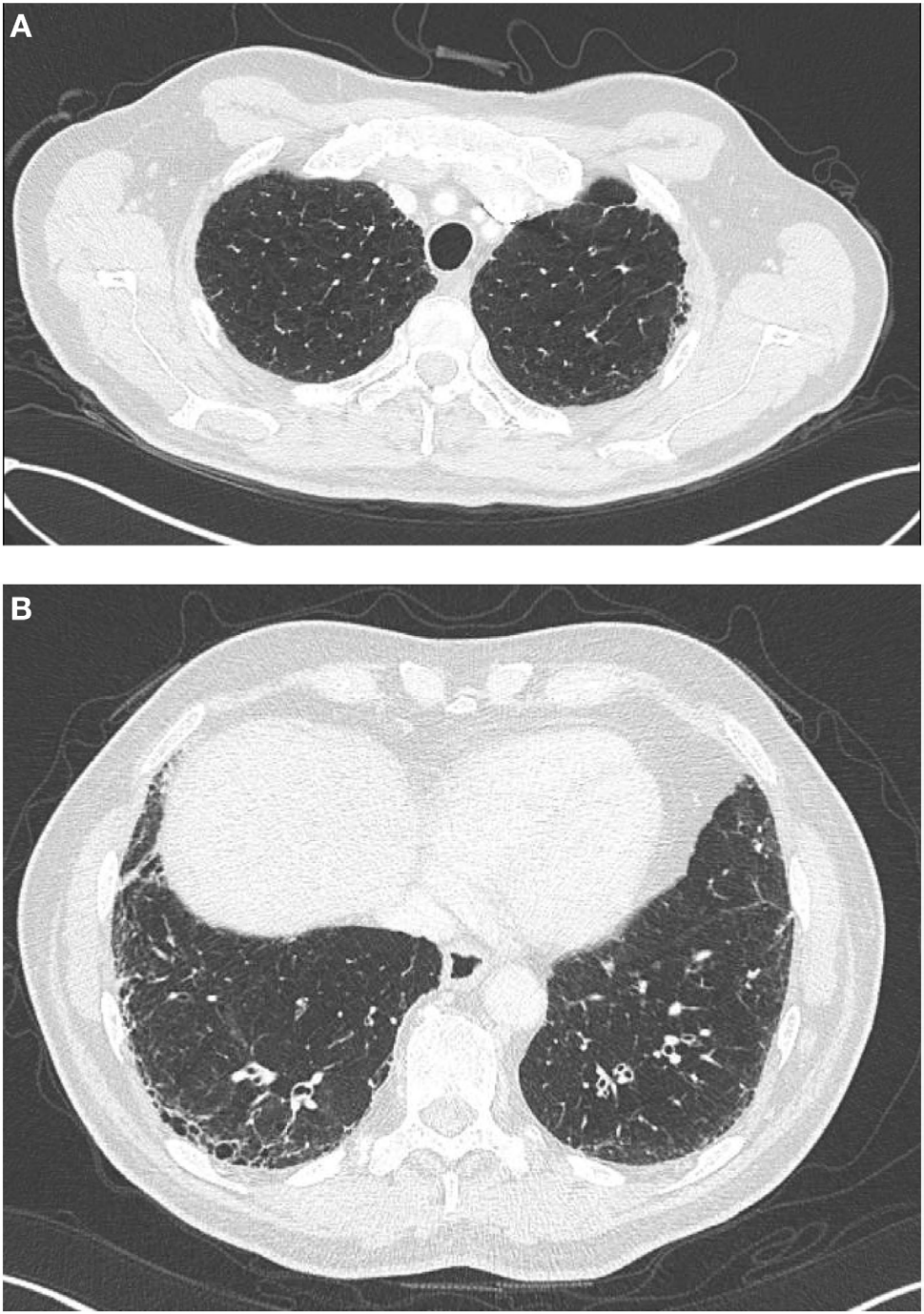
High-resolution computed tomography findings in a patient, with combined pulmonary fibrosis and emphysema. Centrilobular emphysema is observed on apical views **(A)** and basilar predominant sub-pleural reticulation and honeycombing characteristic of UIP is observed on basilar views **(B)**.

**Figure 2:**
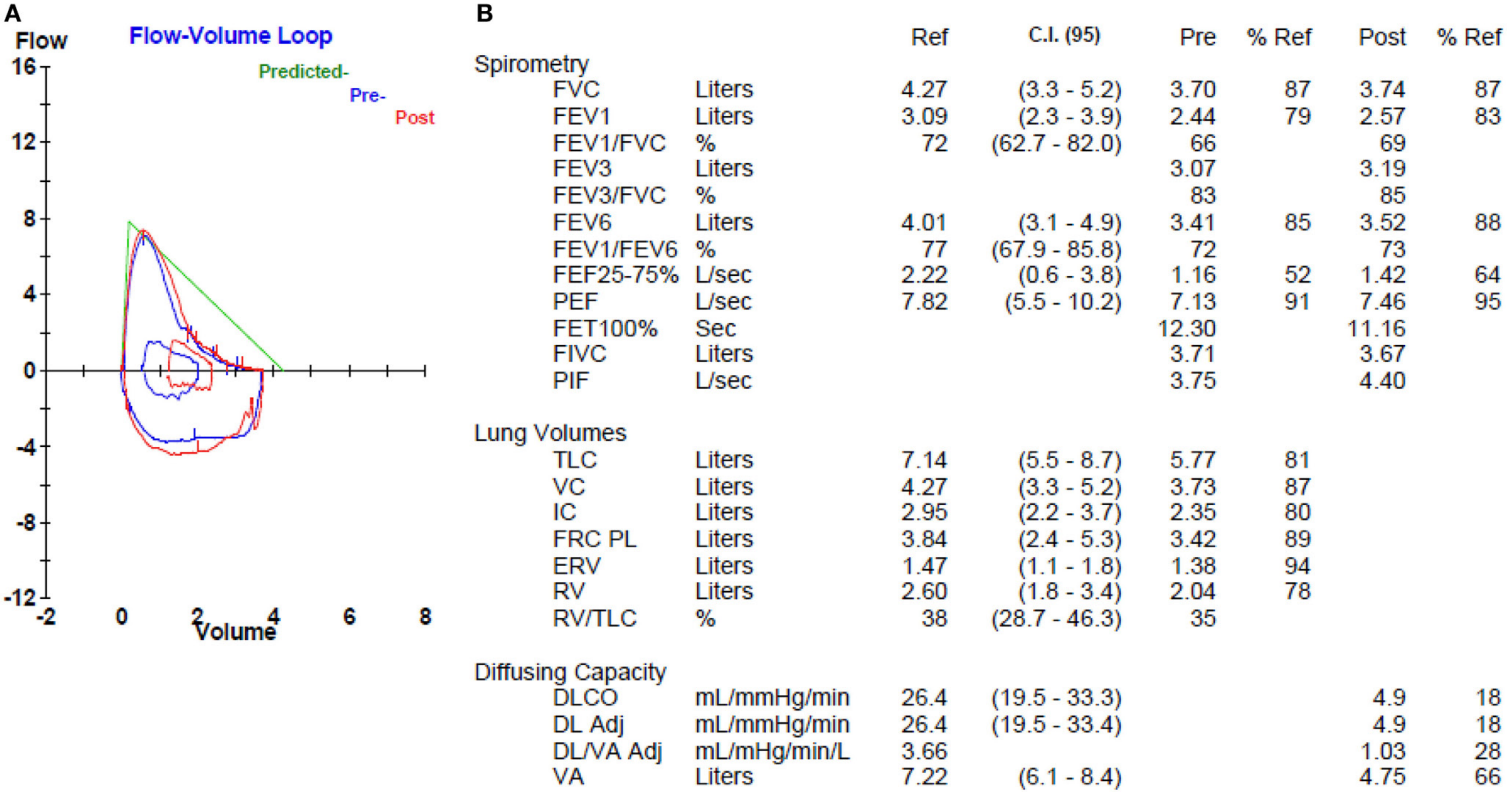
Pulmonary function testing in a patient with combined pulmonary fibrosis and emphysema. Flow volume loop **(A)** shows mild obstructive morphology, but normal spirometry **(B)**. Lung volumes **(B)** are normal. Diffusion capacity **(B)** is markedly reduced.

Roughly 70–80% of individuals with IPF endorse a history of cigarette smoking, which has long been an established IPF risk factor (17–20). Not surprisingly, about 30% of IPF patients have concurrent pulmonary emphysema (21, 22), including 8–27% with ≥10% emphysematous involvement throughout the lungs (Figure 1) (22, 23). The syndrome of combined pulmonary fibrosis and emphysema (CPFE) has recently gained recognition as a potentially unique IPF phenotype (24). Individuals with CPFE tend to be males with an extensive smoking history and increased oxygen requirement (21–24). Pulmonary function testing in these individuals often shows relatively preserved total lung capacity and forced vital capacity (FVC), with a disproportionate reduction in diffusion capacity (DLCO) (Figure 2) (21, 22, 24). These physiologic hallmarks of CPFE likely reflect the opposing impact of parenchymal fibrosis and parenchymal destruction on airflow and lung volumes, along with additive impact on gas exchange.

High-resolution computed tomography (HRCT) is part of the routine diagnostic evaluation of all patients with suspected IPF (4), and routine semi-quantitative assessment of emphysematous involvement may help readily identify those with CPFE once a diagnostic consensus is established. The recognition of CPFE has potential management implications. Some studies suggest that CPFE is associated with reduced survival (21, 23, 25), but others have not replicated this observation (22, 26). Paradoxically, patients with CPFE appear to have a slower rate of FVC decline, perhaps due to the impact of emphysema on the manner in which FVC reflects progressive fibrosis (27). An increased prevalence of pulmonary hypertension (PH) has also been demonstrated among those with CPFE (22, 23), which may also impact survival (23, 24). Treatment of patients with IPF and CPFE with inhaled long-acting anti-cholinergic, inhaled long-acting beta-agonist and/or inhaled corticosteroids is of unclear benefit (21, 24). We believe that clinicians should consider the addition of these therapies according to chronic obstructive pulmonary disease consensus guidelines (28).

### Lung Cancer

Compared to those in the general population, individuals with IPF have a nearly 5-fold increased risk of developing lung cancer, with 3–22% of patients affected and an estimated incidence of 11 cases per 100,000 person-years (29–32). The annual lung cancer risk also appears to rise in the years following IPF diagnosis (32), which was supported by an autopsy study that identified concurrent pulmonary malignancy in nearly 50% of cases with histologic UIP (33). The strong link between IPF and cigarette smoking history (17) likely explains a portion of the increased lung cancer risk, as the overwhelming majority of patients with IPF who develop lung cancer endorse such a history (32–34). The higher prevalence of lung cancer among those with CPFE compared to lone IPF also supports this observation (34–36). Most studies show squamous cell carcinoma to slightly predominate over adenocarcinoma (37), while a recent investigation of IPF-related adenocarcinomas demonstrated a high frequency of bronchiole-associated markers in IPF cases compared to non-IPF controls, suggesting that these tumors may arise from abnormally proliferating bronchioles in areas of honeycomb cyst (38).

Survival among those with IPF and comorbid lung cancer is poor (34, 39) and often stems from malignancy-related clinical deterioration, as similar rates of pulmonary function decline have been demonstrated in those with and without comorbid lung cancer (34). While surgical resection of early stage lung cancer may be curative, IPF severity and disease trajectory must be taken into consideration, given an increased risk of postoperative morbidity and mortality (40, 41). Surgical resection of lung cancer appears to increase the risk of acute exacerbation in patients with IPF, which has been reported in 7–32% of patients (42–45). Acute exacerbation and clinical deterioration have also been described in patients with IPF undergoing treatment with chemotherapy (46, 47) and radiation therapy (48), underscoring the importance of patient education and risk/benefit analysis in those with inoperable lung cancer. Recent studies suggest that the anti-proliferative effects of pirfenidone and nintedanib may synergize with current chemotherapeutic regimens, but additional research is needed (37, 49, 50).

As for emphysema, HRCT serves as a reasonable modality for lung cancer screening, but many HRCT protocols still perform non-contiguous imaging, which can miss early nodules and other local changes; contiguous imaging is required (4). Most lung cancers identified are incidental, with a large minority having a potential surgical cure (34, 39). As such, we believe clinicians should consider annual lung cancer screening with low-dose computed tomography (51) in high-risk patients with IPF, including those with CPFE and/or longstanding smoking history. Clinicians should also maintain a low threshold for repeat imaging in those who experience clinical worsening despite stable pulmonary function or develop symptoms atypical for IPF.

### Obstructive Sleep Apnea (OSA)

Preliminary studies suggest that OSA may be an underappreciated comorbid condition in those with IPF. Three investigations have shown OSA to be present in 58–88% of patients with IPF, with up to 68% having moderate-to-severe OSA based on an apnea–hypopnea index ≥15 events/hour (52–54). In addition, severe sleep apnea was recently shown to be strongly associated with ischemic heart disease in patients with IPF (54). Despite this potential high prevalence, few IPF patients are evaluated for OSA. A retrospective investigation of IPF patients showed that less than 3% of patients were referred for polysomnography (55). Untreated OSA can result in nocturnal hypoxemia, the presence of which was recently shown to predict worsened survival in patients with IPF (56). Nocturnal hypoxemia strongly correlates with an increased right ventricular systolic pressure (RVSP) (56), which may reflect PH (57).

As with the general population, moderate-to-severe OSA is generally treated with continuous positive airway pressure (CPAP). CPAP initiation has been shown to improve quality of life measures and sleep instruments in those with IPF and comorbid moderate-to-severe OSA, though CPAP non-adherence was common (58). The optimal tool for OSA screening in patients with IPF has yet to be determined, as commonly utilized OSA screening tools, including the Epworth sleepiness scale and Sleep Apnea Scale for Sleep Disorders Questionnaire, did not differentiate IPF patients with and without OSA in a recent prospective investigation (52). Until an effective screening tool is established, clinicians should maintain a low threshold for polysomnography referral in patients with IPF.

## Cardiovascular Comorbidities

### Coronary Artery Disease (CAD)

Idiopathic pulmonary fibrosis and CAD share several risk factors, including increasing age, male gender, and smoking history. Among the largest retrospective studies conducted to date, the estimated prevalence of CAD in those with IPF ranges from 4 to 25% (59–63). A prevalence of up to 68% was described in a cohort of 73 IPF patients who underwent cardiac catheterization as part of a lung transplant work-up (64). Among these individuals, 18% of patients had significant CAD, defined as >50% stenosis of a major coronary vessel on cardiac catheterization or need for percutaneous coronary intervention. Longitudinal analyses have suggested that 7% of patients will develop CAD in the years following IPF diagnosis (63) and that such patients have a 3-fold higher risk of experiencing acute coronary syndrome compared to non-IPF control subjects (61).

The U.S. Preventative Services Task Force concluded that there was insufficient evidence to recommend for or against routine CAD screening in asymptomatic, high-risk individuals in the general population (65). Among symptomatic patients, the American Heart Association suggests that cardiac CT may be a reasonable modality for CAD screening, as the presence of coronary calcifications is a reliable predictor of CAD (66). Because HRCT is recommended for all patients with IPF, the assessment of coronary artery calcification may provide a reliable tool for non-invasive CAD screening in this high-risk population. A study of 57 patients with IPF who underwent cardiac catheterization showed that the presence of moderate-to-severe coronary calcifications had a sensitivity and specificity of >80% for detecting CAD (67). Because significant CAD has been associated with worse survival in patients with IPF (64), clinicians should consider a cardiology referral in patients with angina or moderate-to-severe coronary calcifications on HRCT.

### Pulmonary Hypertension

Pulmonary hypertension defined as mean pulmonary artery pressure (mPAP) ≥25 mm Hg (68), commonly complicates IPF, especially as the disease progresses. The true prevalence of PH in those with IPF is difficult to ascertain, as estimates vary widely based on case finding methodology and the IPF population under consideration. A PH prevalence as low as 3% has been reported in patients with IPF using insurance claims data (62) and as high as 84% using transthoracic echocardiogram (TTE) (69). A prevalence of 29–46% has been reported in studies utilizing right heart catheterization, which remains the gold standard for PH detection (60, 68, 70–75). However, these studies may overestimate the true prevalence, as the majority of patients included in these studies underwent right heart catheterization as part of a lung transplant evaluation, suggesting that many had advanced disease.

Pulmonary hypertension should be suspected in patients with dyspnea or oxygen desaturation out of proportion to pulmonary function, disproportionately low DLCO, evidence of right heart failure on physical exam, or evidence of pulmonary artery enlargement and/or right ventricular hypertrophy on imaging studies. TTE, which estimates RVSP as a surrogate for mPAP, is perhaps the most commonly utilized modality to screen for PH. Although TTE-estimated RVSP has been shown to correlate poorly with mPAP determined by right heart catheterization (73, 76), an RVSP >35 mm Hg has been shown to have a sensitivity of >85% for detecting PH in patients with IPF (73). Unfortunately, the specificity of this RVSP cut-off is only 29%, so clinicians should expect a large number of false positives if using this threshold for triggering right heart catheterization. The decision to refer a patient for cardiac catheterization when TTE suggests the presence of PH should be made on a case-by-case basis.

There are currently no approved therapies for the treatment of PH in patients with IPF and the last decade has seen a disappointing number of negative clinical trials using vasodilator therapies. Several studies have investigated the use of PH therapies for IPF in general (regardless of the presence of PH) and failed to demonstrate efficacy in slowing IPF progression (77–79), and did not alter cardiovascular hemodynamics in those with concurrent PH (80, 81). A small trial of ambrisentan in patients with IPF and right heart catheterization-proven PH (NCT00879229) was stopped after a parent trial of ambrisentan showed no benefit in the subgroup of IPF patients with known PH (78). A similar trial of riociguat (NCT02138825), a soluble guanylate cyclase stimulator, was also terminated after interim analysis showed that those in the intervention arm had an increased risk of death and other serious adverse events.

The phosphodiesterase-5 inhibitor sildenafil was studied in patients with advanced IPF (defined by a baseline DLCO of less than 35%). While it did not significantly alter the primary functional endpoint of walk distance, it did show improvements in dyspnea score, oxygenation, and quality of life (82). A *post hoc* subgroup analysis of patients with evidence of PH by TTE showed that sildenafil therapy did improve walk distance as well (83). Based on these data, clinical trials investigating sildenafil in combination with anti-fibrotic therapy for patients with IPF-associated PH (NCT02951429, NCT02802345) are currently enrolling. The most recent evidence-based guidelines conditionally recommend against the routine use of sildenafil in patients with IPF until randomized controlled trials provide more definitive data (16).

### Pulmonary Embolism (PE)/Venous Thromboembolism (VTE)

Relatively few studies have assessed the epidemiology and clinical consequences of PE and more broadly VTE in patients with IPF. A study utilizing U.S. insurance claims data suggested that 2.7% of individuals with a diagnosis code for IPF also carried a diagnosis of PE (62). Another U.S. insurance claims-based investigation showed that among decedents with a diagnosis of IPF, 1.7% had concurrent VTE (84). These estimates were supported by a case–control analysis conducted in the U.K., which reported a VTE prevalence of 2% in their IPF population, which was 2-fold higher than that observed among non-IPF control subjects (61). Danish investigators showed that individuals previously diagnosed with a VTE were at increased risk of developing incident IIP, suggesting that VTE may be a risk factor for IPF and other IIPs (85).

Longitudinal analyses of patients with IPF suggest that the risk of incident VTE is 3–6 times higher among patients with IPF compared to control subjects, with an estimated 6–9 new events per 1,000 person-years (61, 86). As such, clinicians should maintain a low threshold for PE evaluation in patients with progressive symptoms in the setting of stable pulmonary function metrics. PE should also be excluded in patients with acute or subacute clinical worsening, as this often indicates an acute exacerbation (87). A multi-phase HRCT with and without contrast enhancement should be considered to optimally assess the pulmonary vasculature and parenchyma. Lower extremity duplex ultrasound can be considered for patients with a contrast allergy and in those too unstable to undergo HRCT.

The treatment of PE/VTE requires prolonged blockade of the coagulation cascade, which facilitates clot resolution. The American College of Chest Physicians recommend 3 months of anticoagulant therapy in those with VTE provoked by surgery or other known VTE risk factor. These guidelines also recommend at least 3 months of anticoagulant therapy in patients with a first-time unprovoked VTE, after which time the risk–benefit ratio for extended therapy should be considered (88). This assessment is of particular importance in patients with IPF, as warfarin therapy was shown to increase the risk of death in a general population of IPF patients (excluding those who required anticoagulation for a non-IPF indication) (89). Warfarin therapy has also been linked to worse outcomes in uncontrolled studies, including a recent *post hoc* analysis of IPF clinical trial datasets (90, 91). These findings raise the question of whether warfarin therapy should be used in patients with IPF who require anticoagulation for comorbid diseases (e.g. PE, atrial fibrillation). More research is needed to determine the optimal therapy and duration of therapy for patients with IPF with an indication for anticoagulation.

## Gastrointestinal Comorbidities

### Gastro-Esophageal Reflux (GER)

Gastro-esophageal reflux is another common comorbid condition in patients with IPF (92), but the true prevalence of GER in patients with IPF is difficult to ascertain. Several large epidemiological studies have suggested a prevalence of 30–50% (19, 93, 94), but studies that utilized esophageal pH monitoring suggest that GER may affect over 80% of individuals with IPF (95, 96). Complicating estimates further is the fact that some individuals have silent GER (95) and other primarily non-acid GER (97).

The ideal modality for diagnosing GER remains unclear. While all patients should be screened for GER-associated symptoms, including heartburn, choking, and regurgitation, symptom-based screening has a low sensitivity for detecting pathologic GER (95, 98). Fluoroscopic barium swallow testing can detect GER and microaspiration, but this also suffers from poor sensitivity (99, 100). Gastro-esophageal scintigraphy can detect GER with 80% sensitivity but is not a widely available (101). Esophageal pH monitoring remains the gold standard for diagnosing acid GER, with a reported sensitivity and specificity of over 80% (102, 103). Recent studies suggest that multi-channel intraluminal esophageal impedance testing may be a superior modality for detecting both acid and non-acid GER, but this modality is not widely available at present (103, 104).

It has been hypothesized that GER may contribute to the progression of IPF in some patients. Several studies, with mixed results, have explored the influence of anti-acid therapy on IPF disease course. GER therapy was associated with improved survival in a retrospective, multi-center cohort analysis (19) and less pulmonary function decline in a *post hoc* analysis of three clinical trial datasets (105). These findings were not replicated in a recent *post hoc* analysis of a separate clinical trial dataset (106). Additional data evaluating the efficacy of both medical and surgical GER therapy in patients with IPF are expected in the near future as two phase II clinical trials are underway (NC02085018; NCT01982968).

### Hiatal Hernia (HH)

A likely contributor to the high prevalence of GER in patients with IPF is HH, which has been described in 40–53% of patients with IPF (107, 108). While an increasing degree of HH is likely to result in GER symptoms, mild HH can be asymptomatic (108). HH can be identified on HRCT mediastinal views and does not require dedicated imaging. HH treatment largely focuses on GER-associated symptom control, but surgical correction should be considered in patients with refractory symptoms. A retrospective investigation of patients awaiting lung transplantation showed that Nissen fundoplication was well tolerated and was associated with stabilization of oxygen levels in this patient population (109).

## Endocrine/Metabolic Comorbidities

### Hypothyroidism

While 1–2% of men and 5–9% of women in the general population carry a diagnosis of hypothyroidism (110–112), a recent case–control analysis demonstrated a substantially higher prevalence among individuals with IPF, with 13% of men and 28% of women affected (20). Furthermore, those with combined IPF and hypothyroidism had reduced survival compared to those with IPF alone. The biology underpinning an increased prevalence of hypothyroidism in IPF remains unclear, but because autoimmune thyroiditis is the most common cause of hypothyroidism in developed nations (110–112), aberrant immune activation in IPF may play a role.

### Diabetes Mellitus (DM)

Case–control analyses performed in Japan, Mexico, and the U.K. estimated the prevalence of DM type 2 to be 10–33%, among individuals with IPF, which was significantly higher than that of matched control populations (18, 93, 113). These findings persisted after exclusion of individuals treated with systemic corticosteroid therapy, which is known to alter glucose levels (18, 93). Outcome data were not reported in these studies, so it remains unclear whether the presence of DM influences survival in patients with IPF.

## Mental Health Comorbidities

### Depression and Anxiety

Idiopathic pulmonary fibrosis progression commonly manifests as worsening dyspnea, declining pulmonary function, and hypoxemia. While these manifestations undoubtedly impact quality of life, they have been shown to correlate with depression and anxiety (114–116). Despite these findings, few epidemiologic studies of mental health comorbidities have been performed in IPF. While 3.4% of patients with a diagnosis code for IPF also had a diagnosis code for depression in an investigation of insurance claims (62), IPF cohort studies suggest a prevalence of 12–49% (115, 116). Anxiety was shown to affect approximately 10% of IPF patients in a single center study (116).

A small IPF cohort study based in Europe suggested that increasing duration of disease correlates with standardized depression scores (117), while an ILD cohort study based in Australia showed that an increasing number of comorbid conditions also correlates with increasing depression scores (116). The impact of depression and anxiety on outcomes remains unclear in IPF. In addition, the ideal screening tool to detect depression and anxiety in this patient population has yet to be validated.

## Conclusion

Idiopathic pulmonary fibrosis remains a devastating diagnosis for patients and their families, and its management requires a multi-pronged approach. Comorbidity assessment and management is a cornerstone of comprehensive management of IPF and we have reviewed the most commonly associated comorbidities that clinicians should consider (Table 1). Aggressive management of comorbidities is promoted by IPF centers of excellence across the country and may explain some of the improved survival associated with these centers (118). We strongly believe that proper comorbidity assessment and management can improve quality of life and has the potential to improve patient survival in IPF.

**Table 1:** Common comorbidities in patients with idiopathic pulmonary fibrosis.

Comorbidity	Prevalence (%)	Outcome association
Emphysema	8–34	Mixed results; may increase mortality risk Increased prevalence of pulmonary hypertension (PH)
Lung cancer	3–22	Increased mortality risk Surgical resection may increase mortality and acute exacerbation risk Chemotherapy may increase acute exacerbation risk
Obstructive sleep apnea	58–88	May increase mortality risk through worsening nocturnal hypoxemia
Coronary artery disease	4–68	May increase mortality risk
PH	3–84	Increases mortality risk
Pulmonary embolism/venous thromboembolism	2–3	Treatment with anti-coagulation may increase mortality risk
Gastro-esophageal reflux	30–80	Mixed results; treatment with antacid therapy may improve survival and reduce disease progression
Hiatal hernia	40–53	Surgical correction may improve survival and stabilize oxygenation in patients awaiting transplant
Hypothyroidism	13–28	May increase mortality risk
Diabetes mellitus type 2	10–33	Unknown
Depression	12–49	Unknown
Anxiety	10	Unknown

## Author Contributions

JO and HC contributed to the conception and writing of this review. Both authors have reviewed and approved the submitted work.

## Conflict of Interest Statement

JO and HC have no relevant conflicts to disclose related to the submitted work.
